# Improving Cross-Sector Collaborations between Healthcare and Housing: Challenges and Strategies Identified by Unhoused People with Complex Health Needs

**DOI:** 10.1007/s11524-025-00966-z

**Published:** 2025-03-24

**Authors:** Emmy Tiderington, Nora Sullivan, Michael Yedidia, Joel C. Cantor

**Affiliations:** 1 School of Social Work, Rutgers University, New Brunswick, NJ 08901 USA; 2 Center for State Health Policy, Rutgers University, New Brunswick, NJ 08901 USA

**Keywords:** Homeless, Health, Qualitative, Cross-sector

## Abstract

There is growing recognition of the need for collaboration between the healthcare and housing sectors to address the needs of people experiencing homelessness. This study explores how these cross-sector collaborations can be improved from the perspective of those with histories of homelessness and complex health needs. In-depth, semi-structured qualitative interviews (*N* = 23) were used to (1) understand the challenges faced by people with complex health needs when navigating services at the intersection of healthcare and housing and (2) identify strategies for improving these services. While some participants reported accessing cross-sector services, many found those efforts to be lacking and ineffective. Participants reported receiving support with healthcare needs from housing providers more frequently than assistance with housing needs from healthcare providers. They described challenges related to provider turnover, perceived stigma and discrimination, and insufficient resources. Proposed solutions included modernizing and centralizing care, providing an effective balance of in-person and virtual offerings with an emphasis on in-person services, and improving provider sensitivity to reduce stigma against service recipients. These findings align with existing research on cross-sector collaborations in other fields and highlight the need for comprehensive, compassionate care tailored to the unique needs of people experiencing homelessness. The study also underscores the urgent need for more effective implementation and evaluation of these cross-sector efforts to improve outcomes for this vulnerable population.

## Introduction

The bidirectional relationship between housing and health has been well-documented [1–3]. Research has shown that housing is a crucial social determinant of health and that health status can impact one’s housing stability [4]. Within academic and practice communities, there is growing recognition of the need for collaboration between the housing and healthcare sectors and that a siloing of these services can be detrimental to people experiencing homelessness (PEH) [5]. Policymakers and practitioners have then increasingly advocated for better integration between housing and healthcare to adequately meet the needs of those who are unhoused [2], and hospitals and homeless service agencies now face new incentives to collaborate [6].

Existing models of cross-sector collaboration have demonstrated possible routes forward, but not all such efforts have been informed by those who are arguably most impacted—individuals with histories of homelessness and complex health needs. Models have included medical respite centers for PEH which have achieved positive outcomes by providing a non-hospital place for recovery from illness or injury [7]. Another cross-sector strategy for addressing healthcare needs for unhoused individuals is street medicine, which targets healthcare provision for PEH. Limited research on the topic has shown that it holds great promise for improved health outcomes for PEH [8]. Significant efforts to integrate healthcare and housing services emerged amidst the COVID-19 pandemic when it was recognized that PEH were at greater risk of contracting the virus due to the impracticality of public health guidelines to shelter-in-place for those without housing [9]. Other initiatives have included housing interventions that incorporate services that address the health needs of individuals with a history of homelessness, such as permanent supportive housing [10]. These and other efforts to integrate health and housing services show promising outcomes for PEH, but further implementation and evaluation are needed to understand their efficacy. Several existing cross-sector efforts are in place in New Jersey and are the subject of another component of this study [11]. Two examples of these efforts are the incorporation of healthcare in permanent supportive housing initiatives and collaboration between healthcare and housing programs on outreach to unhoused people.

A failure to effectively integrate care for PEH has serious consequences, including higher mortality and morbidity rates and earlier death rates for this group when compared to the general population [12, 13]. Evidence shows that these trends are worsening and require significant policy intervention [14]. Homelessness disproportionately impacts marginalized groups such as Indigenous peoples and African Americans [15]. Additionally, identity-based discrimination has been shown to create mistrust in medical systems [16], and this is particularly true among PEH [17].

The present study explores how cross-sector collaborations can be improved, drawing on the voices of people with lived experience of homelessness and significant healthcare needs in the state of New Jersey. Incorporating the perspectives of people with lived experience is a novel and necessary contribution to the current literature on cross-sector efforts. This study sought to (1) understand the challenges faced by people with complex health needs when navigating services at the intersection of healthcare and housing and (2) identify strategies for improving services at the intersection of healthcare and housing. The pursuit of this data serves not only to capture a snapshot of the reach of cross-sector services in New Jersey, but also to identify existing challenges faced by PEH navigating healthcare and housing and ways that cross-sector programs might resolve those concerns.

## Methods

To achieve these aims, qualitative methods were used. We worked with a community partner familiar with homeless service providers across the state to identify a purposive sample (*N* = 23) of individuals who were currently or had recently experienced homelessness and who had dealt with complex needs within the healthcare system. Participants were drawn from seven counties (of 21) across the state to ensure variability in potential exposure to cross-sector efforts. Prospective participants were contacted through flyers distributed to homeless service partner organizations and posted in public spaces. Prospective participants who saw the flyer called a study phone number and spoke with the interviewer to determine eligibility for the study according to the inclusion criteria (i.e., were currently or had recently experienced homelessness and had dealt with complex needs within the healthcare system). Alternatively, some participants were identified by service provider staff based on known alignment with study criteria.

In-depth, semi-structured interviews were conducted by phone and were on average 1 h in length. The interviewer was a doctoral student research assistant who identified as a White, cis-gender woman with no personal experience of homelessness. The second coder was the lead qualitative researcher on the study, who identifies as a White, cis-gender woman with experience working in homeless services. During the interview, participants were asked a series of questions regarding their experiences receiving housing and healthcare, as well as their awareness of and any experiences with cross-sector services. Participants received a $50 gift card in recognition of their time and effort. Participants were a mixture of current clients of homeless service organizations and those who received word of the study through word-of-mouth. While most participants’ housing situations were fluid, at the time of the interviews, 9 people were currently housed and the remaining 14 were unhoused (unsheltered or living in a shelter, hotel/motel, or a car).

Open coding was used to identify preliminary themes in the first 11 interviews while data collection was ongoing [18]. These themes were then presented to the larger research team for discussion. Following the collection of the remaining interviews, an additional three interviews were coded by the second author to identify any new themes that emerged from these new data and examine whether the preliminary themes held up in the additional interviews. To facilitate a process of consensus coding, the first author reviewed the themes identified by the second author for accuracy and consistency with the interview data. Once consensus was reached, the second author then proceeded to code the remaining nine interviews.

Strategies for rigor were maintained throughout the entirety of the research process [19]. An in-depth audit trail was kept from the start to the completion of the study. The interviewer also recorded memos following each interview to retain major themes, reduce bias, and increase reflexivity [20]. Final themes were reviewed and discussed with the full study team and presented to the study’s stakeholder group as a form of member-checking.

## Results

Thematic analysis identified several challenges that PEH reported experiencing related to cross-sector collaboration and potential strategies for addressing these challenges. Table 1 describes the characteristics of interview participants.

**Table 1:** Participant demographics (*N* = 23)

Characteristics	Percentages and means (SD)
Age (M, SD)	45.8 (11.8)
Age (range)	23–67
Sex (% female)	30.0%
Race and ethnicity	
Caucasian/White	30.0%
African-American/Black	48.3%
Hispanic	8.7%
Multi-racial	8.7%
Other	4.3%
Highest level of education completed	
Less than HS	21.7%
Completed HS or equivalent	34.8%
Completed HS, some college	26.1%
Completed a college degree or higher	17.4%
History of full-time employment	69.6%
Most recent period of employment (M, SD in years)	6 (10)
Years since most recent period of employment (range)	0–39
Longest period of employment (M, SD in years)	6.8 (4.0)
Longest period of employment (range in years)	1–18
Receiving public assistance/entitlement benefits	
Yes	65.2%
No	34.8%
Currently enrolled in Medicaid/NJ Family Care	
Yes	65.2%
No	34.8%
Parent of children	
Yes	78.3%
No	21.7%
Total number of children (M, SD)	2.2 (0.8)
Total number of children (range)	0–4
Total time unhoused in lifetime (M, SD in months)	44.4 (50.2)
Total time unhoused in lifetime (range in months)	1–144
Chronic health conditions	
Yes	91.3%
No	8.7%
Mental health conditions	
Yes	78.3%
No	21.7%
Issues with drugs/alcohol	
Yes	34.8%
No	65.2%

### Challenges in Accessing Needed Supports

#### Personal Priorities Not Always Aligned with System Priorities and Resources

One of the primary challenges faced by participants of this study was difficulty navigating simultaneous needs, particularly when they competed with one another. This navigation was made even more difficult when the person’s personal priorities were out of alignment with the existing structure or apparent aims of the healthcare and/or housing system. People reported frustrations with these misaligned priorities, and often these frustrations were related to difficulties addressing healthcare concerns amidst the pressing need for housing. One participant who was eligible for benefits through the U.S. Department of Veterans Affairs (VA) had theoretical access to health services and also potentially VA supportive housing but had not sought out these services because he viewed the VA as his healthcare provider: “My immediate concern was housing, not Veterans’ Affairs [healthcare]. My immediate concern was housing, it wasn't my health. My health was secondary” [2354].

Seeking out healthcare when homeless could also result in negative unintended consequences, which demonstrated a different kind of misalignment between personal priorities and system priorities. For example, a pregnant participant was told by healthcare providers that without housing, she would be separated from her child once it was born: “I told them, I'm homeless and they said, well, if you give birth, we will take the baby and won't allow you to go home without an address” [2320].

#### Cross-Sector Experiences Occur, but Seamless Integration between Housing and Health Systems Has Yet to Be Realized

Participants shared instances of existing cross-sector housing and healthcare services, but most of these efforts were either not identifiable to participants as such, or the services were less than effective in addressing participants’ needs.

Often participants reported receiving case management services from their housing or homelessness programs, which at times effectively connected service users to healthcare. Simply receiving logistical support from someone who was familiar with navigating these services led to a greater sense among participants that their healthcare and housing needs would be met. In addition to coordinating care by setting up appointments and calling providers, some housing programs supported clients by ensuring transportation to and from appointments:

My case manager helped me. She found several doctors and then I just called around to see which one had the quickest available appointment for me to come into and they actually were able to help the first 30 days that I lived in the shelter with transporting me to the doctor. [2321]

At the same time, several people interviewed felt that it was unexpected for a housing program to ask about healthcare, or vice versa. Some participants experienced a negative reaction in this regard, feeling that programs should “stay in their lane” respective to whatever services they were explicitly offering: “I didn't expect them to even ask like, why are you asking [about healthcare] when I need housing?” [2389].

Notably, it was more common for individuals in this study to report receiving support with healthcare needs from housing providers than it was to receive assistance with housing needs from a healthcare provider. Of the 23 participants in this study, 13 reported receiving assistance with healthcare from their housing provider, while only eight reported receiving assistance with housing from a healthcare provider. Another eight people received no cross-sector support. And only six participants in this study reported receiving assistance with healthcare from their housing provider *and* assistance with housing from a healthcare provider.

#### Programmatic and Provider-Related Barriers to Services and Integration

A consistent challenge identified by most of the participants in this study was difficulties navigating program expectations and logistical barriers, such as long wait times for appointments, fees for unavoidable appointment cancellations, and long travel times to providers in inconvenient locations. Such barriers would be inconvenient for anyone, but these were particularly detrimental to PEH with complex healthcare needs who were on a limited budget with limited resources.

Provider turnover was another barrier to care which caused not only frustration, but also potential disconnection from necessary services. One example was provided by a patient who was promised a dental procedure by one doctor, but after their provider unexpectedly changed, they were no longer able to receive this service:

The doctor I had met at the hospital has already had a turnover and there's a new doctor. So, all they want to do is pull the tooth, they don't want to help me with my dentures or my implants. But the last doctor said he would, but he never put that in the notes. [2320]

This participant shared feelings of insecurity and shame around the fact that she could not receive this dental procedure. She felt that her teeth were a visual marker of substance use that led to stigma and discrimination as she tried to find employment and access other supports. So, this oversight on the provider’s part was not only an inconvenience, but also meant that many other dominoes in her life failed to fall in the direction that she needed for greater stability.

#### Some Supports Are Positive but Cannot Overcome Systemic Failures

Finally, the study team heard from many interviewees that although there were service providers that they believed meant well and provided some support, the systemic challenges that they faced were simply too great to overcome with existing available resources. Even those who were able to access supports were left with questions about how to meet their housing and healthcare needs with existing resources. As one person put it, “How do you live on $700 with two children?” [2319].

Many people interviewed recognized that accessing services and sharing their circumstances with providers meant opening themselves up to potential stigma and mistreatment that could make their situation worse. For one interviewee, “Health care workers don’t know that I’m homeless. I don’t go and tell them that I’m homeless. I don’t tell them that…” [2362]. The subject of physically appearing homeless came up multiple times, with some people sharing that if they could hide their housing status from healthcare providers, they felt it might lead to better outcomes. This presents obvious challenges for cross-sector initiatives attempting to address housing and healthcare concerns simultaneously—how do you provide support for needs you do not know are there?

Participants with complex housing and healthcare needs were familiar with the experience of being underserved by these systems and often felt passed between the housing and healthcare systems with no meaningful sense of overlap or forward momentum. At times, it felt like they were being shuffled between competing systems, what scholars have described as the “institutional circuit” [21]. As one participant said, “There’s no way out of this loop of hospital and shelter, hospital and shelter” [2340].

### Proposed Strategies for Improving Services

#### Modernizing and Centralizing Care

Participants consistently expressed the need for greater accessibility to services. One of the ways that participants believed this goal could be achieved was by improving the quality and accessibility of the resources and information that people needed to access housing and healthcare supports. They proposed making resources and information more up-to-date, comprehensive, and accessible online. Respondents expressed frustration that many resources were not digitized, and existing online resources were either incomplete or inaccessible: “We are in 2023. We are still living in the 1990s on resources and paper and scanned in PDF.” The same interviewee shared, “I want to create a website that literally has, where you can go and apply for OTA [Office of Temporary Assistance], apply for housing, see where all the food resources are” [2320].

Relatedly, a recurring concern was that even if people were theoretically eligible or “on the list” for supports such as housing vouchers, it was extremely common for those promises to remain unfulfilled. People often felt as though they were unable to connect with “the right person” or otherwise access supports they were told were available due to bureaucratic barriers and inconsistencies:

I know people that have Section 8 housing vouchers, but they don't know who to talk to about getting the housing. And talk to one person and they'll give them the runaround and, you know, they give them numbers to another place. [2340]

These struggles were seen as the result of understaffing, poorly managed resources, and inefficient use of resources, among other things. Across the board, participants felt that “the state” was consistently the only place they could turn for support, but that it was reliably unreliable in attending to their concerns.

#### Prioritizing Hassle-Free In-Person Care, with Virtual Options as Needed

While there was no consensus on whether virtual or in-person care were preferable in every situation, overall, there was a sense that in-person options were necessary to provide the type of in-depth support needed by most PEH navigating healthcare and housing needs, and that in-person care should be a priority. However, it was also important that virtual options remain available as appropriate, so that people were not forced to seek in-person services for support that could easily be accomplished online or on the phone. The COVID-19 pandemic was seen as a time when services changed dramatically, and sometimes for the better, but at times, participants felt that the lingering impacts of COVID were used as an excuse to prioritize virtual services even if that did not best serve the user: “They really don’t want you coming in there to social services. They want you to do everything over the phone and everything online” [2303].

One suggestion for a convenient way to receive meaningful, targeted services, particularly for healthcare, was through specialized housing or health “events” designed to meet the needs of PEH that some participants had encountered in the past, for example, a mobile health clinic event for PEH which would allow them to show up on a scheduled day to receive health services. These events could be held on a recurring basis and in a place that was easily accessible for PEH. These events typically did not require advanced appointments and would be predictable so that someone could plan to attend. Said one participant, “Events is the only way you can see a specialist. You have to have some type of event…All you have to do is just show up. It was easy and it’s more convenient” [2374].

Along with this solution for improved accessibility of in-person options, a common refrain was the unnecessary complication of receiving in-person care that could have been a phone call or virtual interaction:

Well, the thing is you have to go, you have to figure out a trip plan…I gotta schedule out a whole half a day or even a whole day for 10 minutes with the doctor, for him to write a prescription. [2301]

Respondents often shared that services seemed more designed for the provider’s convenience than the participant’s. There was an overall desire for improved sensitivity and creativity from providers and that people could ultimately meet their needs in a way that did not increase their already high level of daily burden and stress.

#### Increased Sensitivity from Providers and Other Efforts to Decrease Stigma against Service Recipients

Overall, one of the most common themes heard throughout interviews was simply that participants wished for supportive and caring treatment from providers and to feel that their care team was doing everything they could do to improve their situation. Unfortunately, this was often not the case. Responses showed a significant need for increased sensitivity from providers working with PEH and that stigma and discrimination in both housing and healthcare settings were regrettably common toward this group: “The staff really doesn’t treat us good, but I feel like I’m an animal sometimes, the way they treat us, and the way that they talk to us” [2303].

Often this discrimination arose in the form of stigma toward perceived drug use; people reported not being able to access needed medication from providers who believed that there was a risk of problematic substance use. “They don’t even want to give you medication. You just suffer because of your situation. If you’re homeless, they really don’t want to give you anything” [2374].

Discrimination based on housing and health status was further exacerbated for people with intersecting marginalized identities. One participant shared, “I get treated differently sometimes because of the fact that I am a black single mother, and I don't have the male counterpart to assist me” [2321]. These experiences, among others, demonstrated the high need for sensitivity from providers working with this population and the immense barriers that individuals face without positive relationships with providers.

## Discussion

Overall, these data show that there are positive directions worth following that could strengthen cross-sector collaborations between the healthcare and housing sectors, but comprehensive cross-sector care is not yet the reality for people with complex health needs and a history of homelessness. Since this study sampled from various communities throughout the state, there was not an expectation that every participant had encountered cross-sector services. Rather, these findings imply that overall cross-sector collaboration in the state is lacking.

This study illustrated a range of challenges for PEH navigating health and housing services and identified a series of proposed solutions to improving the cross-sector collaboration between these entities. People interviewed for this study shared that their priorities did not always align with the priorities of the healthcare and housing systems and that although certain cross-sector services existed, they did not typically meet the entirety of people’s needs. The success of these offerings was limited by programmatic and provider-related barriers, as well as ongoing systemic concerns that could not be simply resolved by a single cross-sector initiative. Proposed solutions included modernizing and centralizing care, providing an effective balance of in-person and virtual offerings with an emphasis on in-person services, and improving provider sensitivity to decrease stigma against service recipients.

These findings are aligned with other studies on cross-sector collaborations in other sectors such as medical-legal and medical-financial partnerships [22, 23], which identify a similar range of challenges and opportunities for implementing healthcare-related cross-sector initiatives. Common trends in related studies include the importance of in-person, on-site services [23] and provider buy-in from compassionate, highly trained professionals who are culturally responsive to the needs of the community being served. These findings are also consistent with literature indicating that tailoring healthcare services to PEH is an important step, and patient preference should be considered in both the format of care received and provider selection [24].

Results from research on the implementation of housing and health partnerships within a public housing authority demonstrate that a cross-sector approach can have meaningful outcomes for low-income participants, if significant barriers to implementation are addressed [25]. These barriers include concerns related to privacy when sharing participant data between housing and healthcare entities, as well as liability concerns and a general lack of resources among programs implementing these services. Related findings have reiterated the potential efficacy of these partnerships in both low-income housing settings [26] and among currently unhoused individuals [27]. This study distinguishes itself from these studies by focusing on the experiences of PEH, rather than service providers. More research amplifying the voices of PEH who have directly participated in existing cross-sector efforts is needed to adequately inform these partnerships to meet their intended goals.

Cross-sector service implementation studies have had promising results and show a variety of potential positive outcomes of these initiatives. Most importantly, cross-sector health and housing partnerships have demonstrated a decrease in negative health outcomes for people with low incomes and PEH [26, 27]. Studies on medical respite services for PEH have shown that offering critical, targeted support for PEH in moments of medical crisis that are sensitive to their particular needs reduces overall hospital usage and improves housing outcomes [7]. Findings such as these show that cross-sector services hold promise for both the health and housing outcomes of people currently underserved by both systems. However, without significant emphasis on the experience of stigma and discrimination felt by many people both within this study’s sample and beyond [16, 17], serious risks abound.

This study distinguishes itself from existing research on cross-sector initiatives by focusing on a general population of PEH. This sampling strategy is unique because it illustrates a range of housing and healthcare needs and experiences from people who may or may not have been targeted by an existing cross-sector initiative. As an understanding of the importance of housing as a social determinant of health grows [1, 28, 29], it is important for research to assess the felt impact of cross-sector efforts on directly impacted individuals. As demonstrated by the present findings, despite growth in cross-sector programming in the state, there was a low level of experience with known cross-sector programming reported by study participants. While participants reported cross-sector activities occurring between housing and healthcare providers at times, they were inconsistent and often did not result in successfully achieving participants’ goals. These findings indicate a need for greater and more efficient cross-sector programming that is consistently attentive to the needs of PEH.

One especially notable finding from this study was that participants were more likely to connect with healthcare through housing providers than with housing through healthcare providers. A potential explanation for this, as described by participants, is the lack of transparency PEH may have about their housing status with their healthcare providers due to the perceived potential of a stigmatizing or discriminatory response. Training healthcare providers to purposefully screen for homelessness using ICD-10 Z-codes and how to empathically respond to individuals who report housing instability is one possible intervention to address this gap [30].

This study is not without limitations—the decision to conduct interviews on the phone may have impacted who participated, since people with less stable circumstances may have faced difficulties contacting the study. Additionally, while the research team felt confident in our screening methods, using an open recruitment flyer meant that some people who did not fit the study criteria may have contacted the study in pursuit of the financial incentive.

While the literature confirming the potential benefit of cross-sector healthcare and housing collaboration is substantial, this study demonstrates the need for a greater understanding of how implementation, format, and program characteristics ultimately influence the healthcare and housing outcomes of participating individuals. It is also crucial that cross-sector efforts address histories of medical mistrust among PEH and people with otherwise marginalized identities to provide effective solutions. In addition to targeted funding and logistical support for cross-sector programming, ongoing research evaluating its challenges and successes will be crucial to effecting positive outcomes for PEH. By including PEH with complex needs in these evaluation efforts, new insights into these limitations and opportunities can be illuminated.

## Data Availability

The participants of this study did not give written consent for their disaggregated interview data to be shared publicly, so due to the sensitive nature of the research supporting data is not available.
